# Redefining drug-induced tremor: a mechanism-based framework for clinical and etiological assessment

**DOI:** 10.3389/fneur.2026.1921752

**Published:** 2026-10-28

**Authors:** Hae-Won Shin

**Affiliations:** Department of Neurology, Chung-Ang University Hospital, Chung-Ang University College of Medicine, Seoul, Republic of Korea

**Keywords:** drug-induced tremor, drug-induced parkinsonism, tardive tremor, mechanism-based approach, dopamine-transporter image

## Abstract

Drug-induced tremor (DIT) encompasses clinically diverse syndromes with different mechanisms, clinical courses, and management needs. This Perspective proposes a mechanism-based approach to guide evaluation and management by integrating tremor phenotype, drug pharmacology, associated neurological findings, and clinical course. The initial assessment considers peripheral adrenergic amplification, dopamine receptor blockade or depletion, and systemic or neurotoxic effects, while allowing drug-specific assessment when mechanisms are less clearly understood, as with valproate and serotonergic antidepressants. These considerations guide treatment adjustment and targeted investigation. Associated cerebellar, neuropathic, cognitive, or systemic findings guide evaluation for neurotoxicity and metabolic abnormalities. When tremor is accompanied by parkinsonian features, clinical follow-up assesses recovery, persistence, or progression. Dopamine transporter (DAT) imaging is considered when uncertainty about coexisting nigrostriatal degeneration would affect management. Assessment also considers tardive tremor, taking into account its clinical features and the timing of symptoms in relation to prolonged drug exposure and withdrawal. By connecting these assessments to investigation, treatment, follow-up, and counseling, the proposed framework complements the Movement Disorder Society tremor classification and provides a practical approach to the heterogeneity of DIT. Prospective validation is needed to determine its clinical utility.

## 1 Introduction: one label, diverse mechanisms

Drug-induced tremor (DIT) is a common clinical problem, but the term encompasses tremor syndromes with substantial mechanistic and prognostic heterogeneity (1). In the 2018 Movement Disorder Society (MDS) consensus classification, tremor is described by clinical features on Axis 1 and etiology on Axis 2, where drug-induced tremor is placed among acquired etiologies (2). This two-axis structure was an important advance because it preserves the clinical phenomenology of tremor while allowing drug exposure to be recognized as an etiological factor (2, 3). However, the etiological label alone does not indicate whether tremor is isolated or accompanied by other neurological or systemic findings, nor does it predict the clinical course. Further assessment is therefore needed to guide investigation, treatment, and counseling.

Beta-2 agonist-associated action tremor, antipsychotic-associated tremor with bradykinesia, and tremor emerging after dopamine receptor-blocker withdrawal illustrate this heterogeneity. Their shared association with drug exposure does not imply a common mechanism or prognosis.

This Perspective proposes a practical approach that combines drug pharmacology and tremor phenotype with clinical course and, when indicated, biomarker findings. Parkinsonian signs direct attention to dopamine-system involvement and possible underlying degeneration.

A central distinction is between persistent drug-induced parkinsonism (DIP) and drug-unmasked degenerative parkinsonism. Drug-unmasked degenerative parkinsonism refers to previously subclinical degenerative parkinsonism that becomes clinically apparent following drug exposure. Persistence alone cannot distinguish these possibilities, because parkinsonian signs may remain after withdrawal despite normal dopamine transporter (DAT) imaging (4, 5).

## 2 Principles of the proposed framework

Similar tremor phenotypes may arise through different mechanisms, and the same drug may produce different clinical presentations. The proposed framework therefore considers tremor phenotype and drug pharmacology together with accompanying findings and the subsequent clinical course.

The assessment addresses three questions: Is tremor isolated or accompanied by other neurological or systemic findings? What pharmacological effects of the suspected drug could account for the presentation? If parkinsonian features are present, would evaluating presynaptic nigrostriatal integrity clarify the diagnosis or alter management? The initial assessment guides investigation and treatment adjustment, while follow-up helps refine the interpretation of the drug’s contribution and any coexisting disorder. Tardive chronology is assessed in parallel because it may overlap with other presentations. The framework operates within the acquired etiological category of the MDS classification and does not introduce additional formal axes. Its purpose is to connect tremor phenomenology with treatment direction, biomarker selection, patient counseling, and research design.

## 3 Initial mechanistic assessment

Assessment begins with tremor phenotype, the timing of drug initiation and dose changes, and accompanying neurological or systemic findings. Peripheral adrenergic amplification, dopamine receptor blockade or depletion, and systemic or neurotoxic effects provide useful mechanistic starting points. These mechanistic categories may overlap, as a single drug may act through multiple mechanisms, and an individual patient’s tremor may reflect more than one mechanism.

Before attributing tremor to a drug, clinicians should determine whether it was present before exposure, including its duration, prior diagnosis, and relevant family history. A temporal relationship to drug initiation or dose escalation, dose dependence when assessable, and improvement after drug adjustment support a drug contribution but do not by themselves distinguish newly induced tremor from drug-aggravated pre-existing tremor. Persistence or progression should prompt reassessment for an underlying or coexisting tremor disorder.

For drugs such as valproate and serotonergic antidepressants, the mechanisms underlying tremor remain incompletely understood, and a shared dopaminergic mechanism has not been established. Their assessment therefore draws on the known pharmacology of each drug and the accompanying clinical findings. Table 1 summarizes the proposed mechanistic assessment and subsequent clinical interpretation. Figure 1 outlines how associated findings guide targeted investigation, treatment adjustment, selective DAT imaging, and follow-up.

**Table 1:** Mechanism-based assessment and clinical interpretation of drug-induced tremor.

Assessment component	Pathway or presentation	Mechanistic basis	Representative drugs	Clinical features	Assessment and management
Initial mechanistic assessment	Peripheral adrenergic effects	Beta-adrenergic amplification of physiological tremor; mechanical-reflex resonance	Beta-2 agonists and related sympathomimetics	High-frequency postural or kinetic tremor; no bradykinesia or rigidity	Review dose and indication; consider substitution or symptomatic treatment when appropriate. DAT imaging is not routinely indicated.
	Dopamine receptor blockade or depletion	Dopamine receptor blockade or dopamine depletion; may involve postsynaptic and circuit-level effects	Dopamine receptor blockers and dopamine-depleting agents; cinnarizine and flunarizine also have receptor-blocking properties	Rest, postural, or mixed tremor within a combined parkinsonian syndrome	Assess clinical course and etiology; consider DAT imaging when indicated
	Systemic or neurotoxic pathway	Drug-specific neurological or systemic effects; accompanying findings guide evaluation	Lithium, amiodarone, tacrolimus, cyclosporine	Postural or kinetic tremor; ataxia, neuropathic findings, or systemic abnormalities may coexist.	Assess toxicity and contributing abnormalities. Select drug concentrations, renal/hepatic function, electrolytes, thyroid tests, or NCS/EMG as indicated. Adjust treatment accordingly.
	Valproate-associated tremor	Mechanism uncertain; altered GABAergic neurotransmission and cerebellar dysfunction proposed	Valproate	Usually postural or action tremor; rest tremor or parkinsonism may occur	Drug-specific management; reassess if parkinsonian signs or an atypical course develops
	Serotonergic antidepressant-associated tremor	Mechanism uncertain; contribution of serotonin–dopamine interactions to tremor remains unclear	SSRIs and selected SNRIs	Often postural or action tremor; other extrapyramidal manifestations may occur	Review drug chronology and associated motor signs; use drug-specific management
Clinical course and etiological interpretation	Reversible DIP	Reversible pharmacological interference with dopamine signaling	Dopamine receptor blockers or dopamine-depleting drugs	Tremor with parkinsonism that resolves after withdrawal or dose reduction	Resolution after drug adjustment establishes reversibility. DAT imaging, when obtained, is generally normal or near-normal.
	Persistent DIP with normal or near-normal DAT imaging	Mechanism remains unresolved	Dopamine receptor blockers; other drugs implicated in parkinsonism in selected cases	Tremor and parkinsonian signs persist or only partially improve despite visually normal DAT imaging	Persistence alone does not establish PD. Interpret imaging with the longitudinal clinical course.
	Drug-unmasked degenerative parkinsonism	Presynaptic nigrostriatal degeneration revealed or exacerbated by drug exposure	Dopamine receptor blockers and other drugs associated with parkinsonism in susceptible patients	Tremor with parkinsonism, often asymmetric, persistent, progressive, or associated with prodromal features	Abnormal DAT imaging supports a presynaptic deficit, not a specific diagnosis. Follow-up and treatment depend on the clinical syndrome.
Additional assessment	Tardive tremor	Proposed postsynaptic receptor hypersensitivity or maladaptive striatal signaling after chronic dopamine receptor blockade	Long-term dopamine receptor-blocking agents	Low-frequency tremor (3–5 Hz), predominantly postural, with possible kinetic and rest components. Persistence or worsening after withdrawal and other tardive manifestations provide additional clues.	Assess tremor phenotype together with exposure history and the course after drug adjustment. Consider DAT imaging when coexisting nigrostriatal degeneration is suspected.

DAT, Dopamine Transporter; NCS, Nerve Conduction Study; EMG, Electromyography; GABA, Gamma-aminobutyric acid; SSRI, Selective Serotonin Reuptake Inhibitor; SNRI, Serotonin-Norepinephrine Reuptake Inhibitor; DIP, Drug-induced Parkinsonism; PD, Parkinson disease.

**Figure 1:**
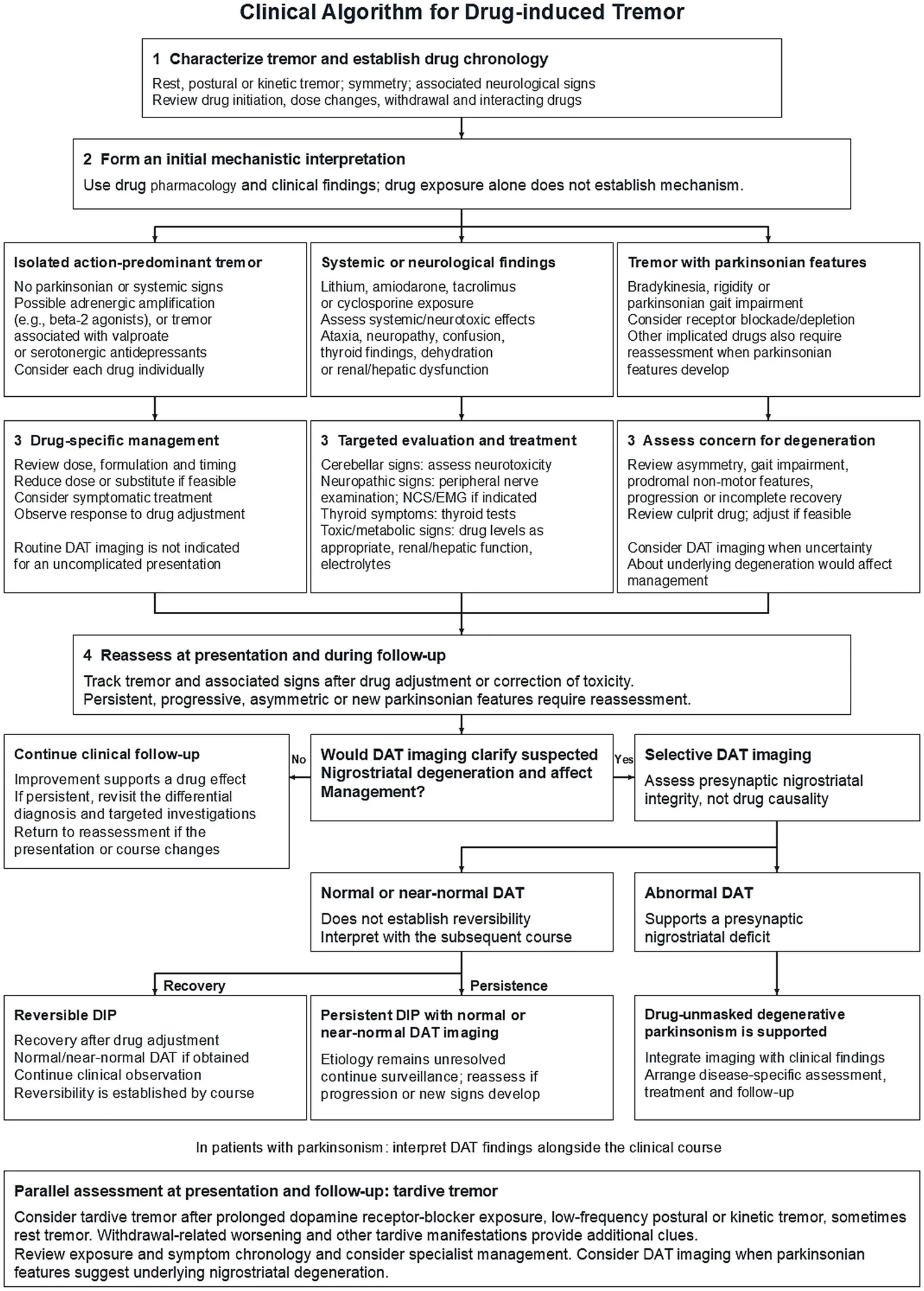
Proposed clinical algorithm for suspected drug-induced tremor. Assessment integrates tremor phenotype, drug chronology, and associated neurological or systemic findings. Drug pharmacology guides the initial interpretation; accompanying findings direct targeted investigation, drug-specific management, or assessment for parkinsonism. These pathways may overlap. Suspected toxicity requires prompt evaluation and treatment. DAT imaging may be considered when uncertainty about coexisting nigrostriatal degeneration would affect interpretation or management. When clinical suspicion is high, imaging may also be considered before the response to drug withdrawal becomes clear. Normal or near-normal findings do not establish reversibility. In patients with parkinsonism, follow-up distinguishes recovery from persistence, while abnormal DAT findings support a presynaptic deficit and, in the appropriate clinical context, drug-unmasked degenerative parkinsonism. Tardive chronology is assessed in parallel because tardive and degenerative syndromes may coexist. DAT, dopamine transporter; DIP, drug-induced parkinsonism; NCS/EMG, nerve conduction studies/electromyography.

When clinical phenotyping remains uncertain, accelerometry and surface electromyography, if available, may help characterize frequency and muscle activation; a frequency shift with inertial loading can suggest a mechanical component. Such findings require clinical interpretation and are not prerequisites for applying the framework (6).

## 4 Tremor outside direct dopamine receptor blockade or depletion

Beta-2 agonists and related sympathomimetics can enhance physiological tremor through peripheral adrenergic effects, including effects on skeletal muscle and mechanical-reflex resonance (7, 8). The typical presentation is a high-frequency postural or kinetic tremor following drug initiation or dose escalation, without accompanying parkinsonian signs. Tremor may improve after dose reduction or substitution of the causative drug. Symptomatic treatment, including propranolol when appropriate, requires consideration of the underlying indication and contraindications.

Lithium, amiodarone, and calcineurin inhibitors such as tacrolimus and cyclosporine can cause tremor and other neurological or systemic adverse effects. Tremor caused by those drugs is usually postural or kinetic (1). Ataxia, dysarthria, or confusion suggests lithium neurotoxicity, particularly when renal impairment or dehydration reduces lithium clearance (9, 10). Amiodarone and calcineurin inhibitors are also associated with other neurological adverse effects such as peripheral neuropathy and cerebellar ataxia (11, 12).

Accompanying neurological or systemic findings guide the selection of investigations. Ataxia, dysmetria or nystagmus suggests cerebellar dysfunction and warrants evaluation for drug-related neurotoxicity. Neuropathic findings may warrant nerve conduction studies and electromyography, while thyroid symptoms warrant thyroid function testing. Cognitive changes, signs of systemic toxicity, renal or hepatic dysfunction, or suspected metabolic abnormalities warrant a review of drug exposure and assessment of relevant drug concentrations, renal and hepatic function and electrolytes, as clinically indicated. Management is guided by the suspected drug exposure and identified abnormalities.

The mechanisms underlying tremor associated with valproate and serotonergic antidepressants remain incompletely understood. Valproate commonly causes postural or kinetic tremor, but rest tremor and parkinsonism can occur (13–15). Proposed mechanisms for valproate-induced tremor include altered GABAergic neurotransmission and cerebellar dysfunction (16, 17). Serotonergic antidepressants may cause tremor and other extrapyramidal manifestations (18). Serotonin–dopamine interactions have been implicated in some of these extrapyramidal effects, but their contribution to antidepressant-associated tremor is less clear (19).

## 5 Tremor within the dopamine system: parkinsonism and tardive presentations

Dopamine receptor blockade or dopamine depletion may cause tremor as part of DIP, accompanied by bradykinesia, rigidity, or gait disturbance (4, 20). Cinnarizine and flunarizine, commonly classified as calcium channel blockers, also have dopamine receptor-blocking properties and can cause parkinsonism with tremor (21). Clinical follow-up after drug adjustment determines whether tremor and accompanying parkinsonian signs resolve, persist, or progress, while DAT imaging, when indicated, assesses presynaptic nigrostriatal integrity.

Reversible DIP is defined by resolution of the parkinsonian syndrome after dose reduction or withdrawal. DAT imaging, when performed, is generally normal or near-normal, consistent with pharmacological interference in dopamine signaling (22, 23). Recovery may be delayed, particularly in older patients or after prolonged exposure (23, 24). A normal scan alone does not establish reversibility. Persistent DIP with normal or near-normal DAT imaging describes a different clinical course. Some patients have residual tremor and other parkinsonian signs after withdrawal despite visually normal scans (5).

Drug-unmasked degenerative parkinsonism refers to previously subclinical degenerative parkinsonism that becomes clinically apparent following drug exposure. Asymmetry, gait impairment, hyposmia, and rapid-eye-movement sleep behavior disorder may suggest underlying neurodegenerative parkinsonism (25, 26). Abnormal DAT imaging supports a presynaptic nigrostriatal deficit, suggesting underlying degeneration unmasked by drug exposure in this clinical setting (22, 23). Progression of parkinsonian signs after withdrawal of the suspected drug further supports this possibility (4). A levodopa response observed during clinical care may contribute to the broader assessment, but tremor response alone is not a defining discriminator in this framework, particularly when receptor blockade continues or dopaminergic treatment is clinically unsuitable.

Tardive tremor is a rare manifestation associated with prolonged exposure to dopamine receptor–blocking drugs. It has been described as a low-frequency tremor (3–5 Hz), most prominent during posture but also present at rest and during voluntary movement. Persistence or worsening after drug withdrawal and accompanying tardive manifestations provide additional clinical clues. Proposed mechanisms include postsynaptic dopamine receptor hypersensitivity and maladaptive striatal signaling, although these hypotheses derive largely from studies of tardive syndromes more broadly (27–29).

## 6 Selective use of DAT imaging

DAT imaging helps assess whether underlying presynaptic nigrostriatal degeneration contributes to the tremor (23, 30). Its use is most appropriate when clinical uncertainty about coexisting degeneration would affect diagnosis, counseling, or treatment. Typical isolated action tremor associated with a plausible drug exposure, or a systemic or neurotoxic presentation without clinical concern for degenerative parkinsonism, generally does not require DAT imaging.

DAT imaging may be helpful when the clinical features outlined in Section 5 suggest underlying nigrostriatal degeneration (25, 26). If tremor persists, further assessment may help determine whether imaging is appropriate in light of accompanying findings and the clinical course. When clinical suspicion is high, imaging may also be considered before the response to drug withdrawal becomes clear.

Normal DAT imaging should be interpreted in conjunction with clinical chronology and longitudinal outcome. Such findings argue against an established presynaptic deficit but do not guarantee complete recovery or exclude subtle dysfunction (5). Conversely, reduced uptake supports presynaptic dysfunction without identifying its specific cause. Thus, DAT imaging informs etiological interpretation, whereas follow-up determines whether the clinical syndrome resolves, persists, or progresses.

## 7 Discussion and future directions

DIT is an umbrella term that encompasses tremor arising through different mechanisms and following different clinical courses. This perspective proposes an approach that distinguishes these presentations using tremor phenotype, drug pharmacology, accompanying clinical findings, and longitudinal outcomes. These distinctions may help guide treatment and the selection of further investigations, including DAT imaging when underlying neurodegenerative parkinsonism is suspected. The aim is to make the diagnosis of DIT more informative for clinical management.

The framework has several limitations. The mechanisms of tremor remain unclear for many drugs, and more than one mechanism may contribute in an individual patient. DAT imaging provides information about presynaptic nigrostriatal function but may not fully explain the tremor. The framework has not yet been validated, and neither the indications for imaging nor the duration of observation after drug withdrawal have been standardized. Its use therefore relies on clinical judgment.

Prospective studies are needed to assess the clinical value of this approach. These studies could record tremor characteristics, accompanying motor and non-motor findings, medication history and dose changes, imaging results when available, and outcomes during follow-up. Outcomes of interest include tremor recovery or persistence, changes in diagnosis and treatment, and the use of imaging. Such studies could assess whether distinguishing these presentations improves treatment selection and the appropriate use of investigations, while complementing the MDS tremor classification.

## Data Availability

The original contributions presented in the study are included in the article/supplementary material, further inquiries can be directed to the corresponding author.

## References

[1] Baizabal-Carvallo JF Morgan JC. Drug-induced tremor, clinical features, diagnostic approach and management. J Neurol Sci. (2022) 435:120192. doi: 10.1016/j.jns.2022.120192, 35220110

[2] Bhatia KP Bain P Bajaj N Elble RJ Hallett M Louis ED . Consensus statement on the classification of tremors. From the task force on tremor of the International Parkinson and Movement Disorder Society. Mov Disord. (2018) 33:75–87. doi: 10.1002/mds.27121, 29193359 PMC6530552

[3] Latorre A Hallett M Deuschl G Bhatia KP. The MDS consensus tremor classification: the best way to classify patients with tremor at present. J Neurol Sci. (2022) 435:120191. doi: 10.1016/j.jns.2022.120191, 35247714

[4] Shin H-W Chung SJ. Drug-induced parkinsonism. J Clin Neurol. (2012) 8:15–21. doi: 10.3988/jcn.2012.8.1.15, 22523509 PMC3325428

[5] Hong JY Sunwoo MK Oh JS Kim JS Sohn YH Lee PH. Persistent drug-induced parkinsonism in patients with Normal dopamine transporter imaging. PLoS One. (2016) 11:e0157410. doi: 10.1371/journal.pone.0157410, 27294367 PMC4905632

[6] Zhang J Xing Y Ma X Feng L. Differential diagnosis of Parkinson disease, essential tremor, and enhanced physiological tremor with the tremor analysis of EMG. Parkinsons Dis. (2017) 2017:1–4. doi: 10.1155/2017/1597907, 28884038 PMC5573102

[7] Larsson S Svedmyr N. Tremor caused by sympathomimetics is mediated by beta2-adrenoreceptors. Scand J Respir Dis. (1977) 58:5–10. 190674

[8] Baker MR Baker SN. Beta-adrenergic modulation of tremor and Corticomuscular coherence in humans. PLoS One. (2012) 7:e49088. doi: 10.1371/journal.pone.0049088, 23185297 PMC3502512

[9] Baek JH Kinrys G Nierenberg AA. Lithium tremor revisited: pathophysiology and treatment. Acta Psychiatr Scand. (2014) 129:17–23. doi: 10.1111/acps.12171, 23834617

[10] Gitlin M. Lithium side effects and toxicity: prevalence and management strategies. Int J Bipolar Disord. (2016) 4:27. doi: 10.1186/s40345-016-0068-y, 27900734 PMC5164879

[11] Orr CF Ahlskog JE. Frequency, characteristics, and risk factors for amiodarone neurotoxicity. Arch Neurol. (2009) 66:865–9. doi: 10.1001/archneurol.2009.96, 19597088

[12] Wagle Shukla A Lunny C Mahboob O Khalid U Joyce M Jha N . Tremor induced by cyclosporine, tacrolimus, sirolimus, or everolimus: a review of the literature. Drugs R&D. (2023) 23:301–29. doi: 10.1007/s40268-023-00428-4, 37606750 PMC10676343

[13] Karas BJ Wilder BJ Hammond EJ Bauman AW. Valproate tremors. Neurology. (1982) 32:428–32. doi: 10.1212/WNL.32.4.428, 6801541

[14] Brugger F Bhatia KP Besag FMC. Valproate-associated parkinsonism: a critical review of the literature. CNS Drugs. (2016) 30:527–40. doi: 10.1007/s40263-016-0341-8, 27255404

[15] Baizabal-Carvallo JF Alonso-Juarez M. Valproate-induced rest tremor and parkinsonism. Acta Neurol Belg. (2021) 121:515–9. doi: 10.1007/s13760-019-01239-8, 31721077

[16] Hamed SA Abdellah MM. The relationship between valproate induced tremors and circulating neurotransmitters: a preliminary study. Int J Neurosci. (2017) 127:236–42. doi: 10.1080/00207454.2016.1181631, 27161592

[17] Paparella G Angelini L De Biase A Cannavacciuolo A Colella D Di Bonaventura C . Clinical and kinematic features of valproate-induced tremor and differences with essential tremor. Cerebellum. (2021) 20:374–83. doi: 10.1007/s12311-020-01216-5, 33200286 PMC8213593

[18] Leo RJ. Movement disorders associated with the serotonin selective reuptake inhibitors. J Clin Psychiatry. (1996) 57:449–54. doi: 10.4088/JCP.v57n1002, 8909330

[19] Lane RM. SSRI-induced extrapyramidal side-effects and akathisia: implications for treatment. J Psychopharmacol. (1998) 12:192–214. doi: 10.1177/026988119801200212, 9694033

[20] Conn H Jankovic J. Drug-induced parkinsonism: diagnosis and treatment. Expert Opin Drug Saf. (2024) 23:1503–13. doi: 10.1080/14740338.2024.2418950, 39419777

[21] Calzetti S Negrotti A. Outcome of drug-induced parkinsonism in the elderly: a permanent nonprogressive parkinsonian syndrome may occur following discontinuation of Cinnarizine and Flunarizine. Ann Pharmacother. (2025) 59:289–93. doi: 10.1177/10600280241263592, 39054800

[22] Brigo F Matinella A Erro R Tinazzi M. [^123^I] FP-CIT SPECT (Da TSCAN) may be a useful tool to differentiate between Parkinson's disease and vascular or drug-induced parkinsonisms: a meta-analysis. Eur J Neurol. (2014) 21:1369–e90. doi: 10.1111/ene.12444 24779862

[23] Shin H-W Kim JS Oh M You S Kim YJ Kim J . Clinical features of drug-induced parkinsonism based on [18F] FP-CIT positron emission tomography. Neurol Sci. (2015) 36:269–74. doi: 10.1007/s10072-014-1945-8, 25231645

[24] Feldman M Marmol S Margolesky J. Updated perspectives on the Management of Drug-Induced Parkinsonism (DIP): insights from the clinic. Ther Clin Risk Manag. (2022) 18:1129–42. doi: 10.2147/TCRM.S360268, 36573102 PMC9789682

[25] Tachibana K Matsuura K Shindo A Matsuyama H Ii Y Taniguchi A . Symptomatic characteristics of Parkinson’s disease induced by neuroleptic drugs, based on a functional neuroimaging diagnosis. Intern Med. (2020) 59:485–90. doi: 10.2169/internalmedicine.2553-18, 32062624 PMC7056363

[26] Aamodt WW Dubroff JG Cheng G Taylor B Wood S Duda JE . Gait abnormalities and non-motor symptoms predict abnormal dopamine transporter imaging in drug-induced parkinsonism. NPJ Parkinsons Dis. (2022) 8:53. doi: 10.1038/s41531-022-00309-8 PMC9051164 35484281

[27] Stacy M Jankovic J. Tardive tremor. Mov Disord. (1992) 7:53–7. doi: 10.1002/mds.870070110, 1348352

[28] Frei K Truong DD Fahn S Jankovic J Hauser RA. The nosology of tardive syndromes. J Neurol Sci. (2018) 389:10–6. doi: 10.1016/j.jns.2018.02.008, 29433810

[29] Hauser RA Meyer JM Factor SA Comella CL Tanner CM Xavier RM . Differentiating tardive dyskinesia: a video-based review of antipsychotic-induced movement disorders in clinical practice. CNS Spectr. (2022) 27:208–17. doi: 10.1017/S109285292000200X PMC9249122 33213556

[30] Diaz-Corrales FJ Sanz-Viedma S Garcia-Solis D Escobar-Delgado T Mir P. Clinical features and 123I-FP-CIT SPECT imaging in drug-induced parkinsonism and Parkinson’s disease. Eur J Nucl Med Mol Imaging. (2010) 37:556–64. doi: 10.1007/s00259-009-1289-4, 19862520

